# Antibody responses to merozoite antigens after natural *Plasmodium falciparum* infection: kinetics and longevity in absence of re-exposure

**DOI:** 10.1186/s12916-019-1255-3

**Published:** 2019-01-30

**Authors:** Victor Yman, Michael T. White, Muhammad Asghar, Christopher Sundling, Klara Sondén, Simon J. Draper, Faith H. A. Osier, Anna Färnert

**Affiliations:** 1 Division of Infectious Diseases, Department of Medicine Solna, Karolinska Institutet, Karolinska University Hospital, 171 76 Stockholm, Sweden; 2 Department of Parasites and Insect Vectors, Institut Pasteur, 25-28 Rue du Dr Roux, 75015 Paris, France; 3 Department of Infectious Diseases, Karolinska University Hospital, 171 76 Stockholm, Sweden; 4 Jenner Institute, University of Oxford, Oxford, OX3 7DQ UK; 5 Kenya Medical Research Institute - Wellcome Trust Research Program, Centre for Geographic Medicine Research-Coast, PO Box 230-80108, Kilifi, Kenya; 6 Centre for Infectious Diseases, Parasitology, Heidelberg University Hospital, Im Neuenheimer Feld 324, 69120 Heidelberg, Germany

**Keywords:** Antibody, Half-life, Longevity, Traveller, Longitudinal, Malaria, *Plasmodium falciparum*, Serology, IgG, Subclass

## Abstract

**Background:**

Antibodies against merozoite antigens are key components of malaria immunity. The naturally acquired antibody response to these antigens is generally considered short-lived; however, the underlying mechanisms remain unclear. Prospective studies of travellers with different levels of prior exposure, returning to malaria-free countries with *Plasmodium* infection, offer a unique opportunity to investigate the kinetics and composition of the antibody response after natural infection.

**Methods:**

Adults diagnosed with *P*. *falciparum* malaria in Stockholm, Sweden (20 likely malaria naïve and 41 with repeated previous exposure during residency in sub-Saharan Africa) were sampled at diagnosis and 10 days and 1, 3, 6, and 12 months after treatment. Total and subclass-specific IgG responses to *P*. *falciparum* merozoite antigens (AMA-1, MSP-1_19_, MSP-2, MSP-3, and RH5) and tetanus toxoid were measured by multiplex bead-based immunoassays and ELISA. Mathematical modelling was used to estimate the exposure-dependent longevity of antibodies and antibody-secreting cells (ASCs).

**Results:**

A majority of individuals mounted detectable antibody responses towards *P*. *falciparum* merozoite antigens at diagnosis; however, the magnitude and breadth were greater in individuals with prior exposure. In both exposure groups, antibody levels increased rapidly for 2 weeks and decayed thereafter. Previously exposed individuals maintained two- to ninefold greater antibody levels throughout the 1-year follow-up. The half-lives of malaria-specific long-lived ASCs, responsible for maintaining circulating antibodies, ranged from 1.8 to 3.7 years for merozoite antigens and were considerably short compared to tetanus-specific ASCs. Primary infected individuals did acquire a long-lived component of the antibody response; however, the total proportion of long-lived ASCs generated in response to infection was estimated not to exceed 10%. In contrast, previously exposed individuals maintained substantially larger numbers of long-lived ASCs (10–56% of total ASCs).

**Conclusion:**

The short-lived nature of the naturally acquired antibody response, to all tested merozoite antigens, following primary malaria infection can be attributed to a combination of a poor acquisition and short half-life of long-lived ASCs. Greater longevity is acquired with repeated infections and can be explained by the maintenance of larger numbers of long-lived ASCs. These insights advance our understanding of naturally acquired malaria immunity and will guide strategies for further development of both vaccines and serological tools to monitor exposure.

**Electronic supplementary material:**

The online version of this article (10.1186/s12916-019-1255-3) contains supplementary material, which is available to authorized users.

## Introduction

Antibodies are critical components of naturally acquired immunity to malaria and are particularly important during the blood stage of the infection where targets include antigens expressed on the surface of the merozoite and the infected red blood cell [1–3]. An understanding of the acquisition and maintenance of the antimalarial antibody response is crucial for improving prospects for successful vaccine development [2, 4, 5] as well as to guide further design of reliable serological tools for transmission surveillance [6, 7]. This requires a detailed characterisation of the kinetics of the antibody response following infection, including estimates of the longevity in individuals of different ages and with different levels of prior malaria exposure [8–10].

The longevity of an antibody response is primarily determined by the generation and survival of long-lived antibody-secreting cells (ASCs) [11–13]. For many viral and bacterial infections, a protective and long-lived response is acquired after a single exposure. Furthermore, the half-life of the antibody response to several common vaccine antigens has been estimated to range from approximately a decade, in the case of tetanus toxoid, to life-long and without decay, in the case of the measles vaccine [14]. However, acquisition of immunity to malaria requires repeated infections [15, 16] and, although protective antibodies are acquired with time [1, 17, 18], the antibody response appears to be short-lived particularly in children [5, 19, 20].

The longevity of long-lived malaria-specific ASCs is difficult to study as they reside in the bone marrow and the secondary lymphoid tissues and are only transiently detectable in peripheral blood following acute infection [21]. However, the number of bone marrow ASCs correlates with circulating antibody levels in both mice experimentally infected with *Plasmodium chaubaudi* [22] and HIV-infected humans [23]. In addition, modelling of longitudinal antibody data from highly malaria-exposed children using a bi-exponential decay model has been shown to allow for estimation of the half-life of malaria-specific IgG antibodies and both short- and long-lived ASCs, as well as their proportional contribution to the response [5]. IgG consists of four different subclasses (IgG_1–4_), each with different sets of effector functions and rates of turnover due to the inherent differences in their biochemical properties. The underlying IgG subclass profile may therefore influence the half-life of the antigen-specific total IgG response [5, 24].

Reliable estimates of decay rates of antimalarial antibody responses require detailed study of the kinetics of the response after infection. However, studies of antibody kinetics in malaria-endemic areas are hampered by difficulties in determining the timing of the latest exposure due to frequent asymptomatic carriage of low-density infections [25] and the continuous risk of reinfection during follow-up, causing boosting of immune responses [5]. This can partially be addressed through studies of controlled human malaria infection (CHMI), in which all of the above are carefully monitored and controlled [26]. However, participants in CHMI trials are typically treated at microscopic patency of blood-stage infection, often before symptoms have occurred [27, 28]. The immune response observed in a CHMI may thus not fully mirror the response following a symptomatic natural blood-stage infection in which parasitaemia is higher and the inflammatory response more pronounced [26].

Studying returning travellers, diagnosed with malaria in malaria-free countries, provides a unique opportunity to investigate the kinetics of the antimalarial immune response after a naturally acquired *Plasmodium falciparum* infection in absence of re-exposure [29, 30]. Furthermore, studying travellers with different levels of prior exposure enables investigation of how exposure affects the acquisition of long-lived ASCs and the overall longevity of the response. Previous studies in travellers have indicated short-lived antibody responses with a higher overall antibody reactivity in semi-immune compared to malaria-naïve individuals [29–31]. However, due to infrequent sampling or lack of longitudinal follow-up, it has not been possible to provide quantitative estimates of antibody decay rates. Moreover, there has been little effort to properly quantify the antigen-specific half-life of the different IgG subclass responses or assess how the level of prior exposure affects the ratio of short- to long-lived ASCs.

Here, we investigate the kinetics of the total IgG, and IgG subclass-specific, antibody response to *P*. *falciparum* schizont extract and eight recombinant vaccine candidate antigens after a naturally acquired infection in travellers followed prospectively after treatment in Sweden. We use mathematical models to compare how the kinetics and the longevity differ with regard to prior malaria exposure and provide quantitative estimates of the decay of the antibody response as well as the relative contribution from short- and long-lived ASCs.

## Materials and methods

### Study population

Adults (*n* = 64) treated for *P*. *falciparum* malaria at Karolinska University Hospital in Stockholm, Sweden, were enrolled in the study upon hospitalisation and followed prospectively for up to 1 year. Venous blood samples were collected at the time of diagnosis, and participants were invited to contribute follow-up samples 10 days and 1, 3, 6, and 12 months after the first sample. A questionnaire detailing country of birth, previous countries of residence, travel history, use of antimalarial prophylaxis, previous malaria episodes, and co-morbidities was administered to each study participant upon diagnosis as well as at the end of follow-up. Additional clinical data were extracted from hospital records. All participants were treated with a full course of artemether-lumefantrine. Sixteen participants, who were hyperparasitaemic (i.e. > 5% parasitaemia), showing signs of severe malaria according to the WHO classification [32], or vomiting at the time of admission, in addition received one to four dose(s) of intravenous artesunate [33]. Fifty-nine successfully cleared the infection following this initial treatment; however, five individuals suffered late treatment failure and presented with recrudescent parasitaemia on days 20–28 following the initial diagnosis. They were successfully treated with a second course of either artemether-lumefantrine or mefloquine, and these cases have been described in detail elsewhere [34]. No new infections were acquired due to new travel during the period of follow-up.

To examine the effect of exposure on the kinetics of the antibody response, and maximise exposure-related differences, we included individuals with either a likely primary malaria infection or with multiple previous infections but excluded individuals with a documented single previous *P*. *falciparum* infection. Out of the 64 individuals, 20 were European natives with no prior history of malaria infection and a reported median cumulative time of residency in an endemic area of 0 years (range 0–3 years, 16 of the 20 participants reported only short-term travel to malaria-endemic areas). These 20 individuals were considered likely primary infected and are referred to as being “previously naïve”. Forty-one participants (39 born in sub-Saharan Africa) reported multiple prior malaria episodes and a cumulative time of residency of more than 13 years in a highly malaria-endemic area (median 25 years; range 13–39). They were considered to have had repeated prior exposure and are referred to as being “previously exposed”. Three European natives had a documented single previous *P. falciparum* infection and were excluded from analyses.

### Antibody assays

An ELISA was used to quantify total IgG levels to schizont extract (PfSE) (3D7 clone) according to a previously described protocol [35]. Multiplex bead-based immunoassays were used to quantify IgG antibody responses, total IgG and subclasses (IgG_1–4_), to eight recombinant *P*. *falciparum* antigens as previously described [8, 36, 37]. Antigens included full-length reticulocyte-binding protein homologue 5 (RH5) (3D7) v2.0 [38] with a C-terminal C-tag [39] (also known as RH5.1), the 19-kDa fragment of merozoite surface protein-1 (MSP-1_19_) [40], and two allelic variants of each of MSP-2 (CH150/9 and Dd2) [41], MSP-3 (3D7 and K1) [42], and apical membrane antigen 1 (AMA-1) (3D7 and FVO) [43]. The assay was performed separately for the detection of total IgG and each of the four IgG subclasses, IgG_1–4_. In addition, a singleplex bead-based immunoassay was used to quantify total IgG antibodies to tetanus toxoid (TTd) according to a previously described protocol [44]. To examine how the magnitude of the response to RH5 compared to the results from previous studies, a subset of the samples (*n* = 82), collected at the peak of the antibody response and towards the end of the follow-up period, was analysed by a validated ELISA previously used for quantification of RH5-specific IgG [45]. Positive and negative controls and a serially diluted standard calibrator were run on each plate. A threshold of seropositivity was defined as the mean reactivity of 20 malaria unexposed controls plus 3 standard deviations. For each antigen, the assay optical density (OD) or median fluorescent intensity (MFI) was converted to a relative concentration in arbitrary units by interpolation from the standard calibrator curve using a five-parameter sigmoidal curve fitting. The antibody levels in arbitrary antibody units are directly comparable only within each assay, i.e. within antigen and IgG subclass. A detailed description of the protocols is presented as supplementary information (Additional file 1: Supplementary methods).

### Statistical analysis and mathematical modelling of antibody kinetics

**Fig. 1:**
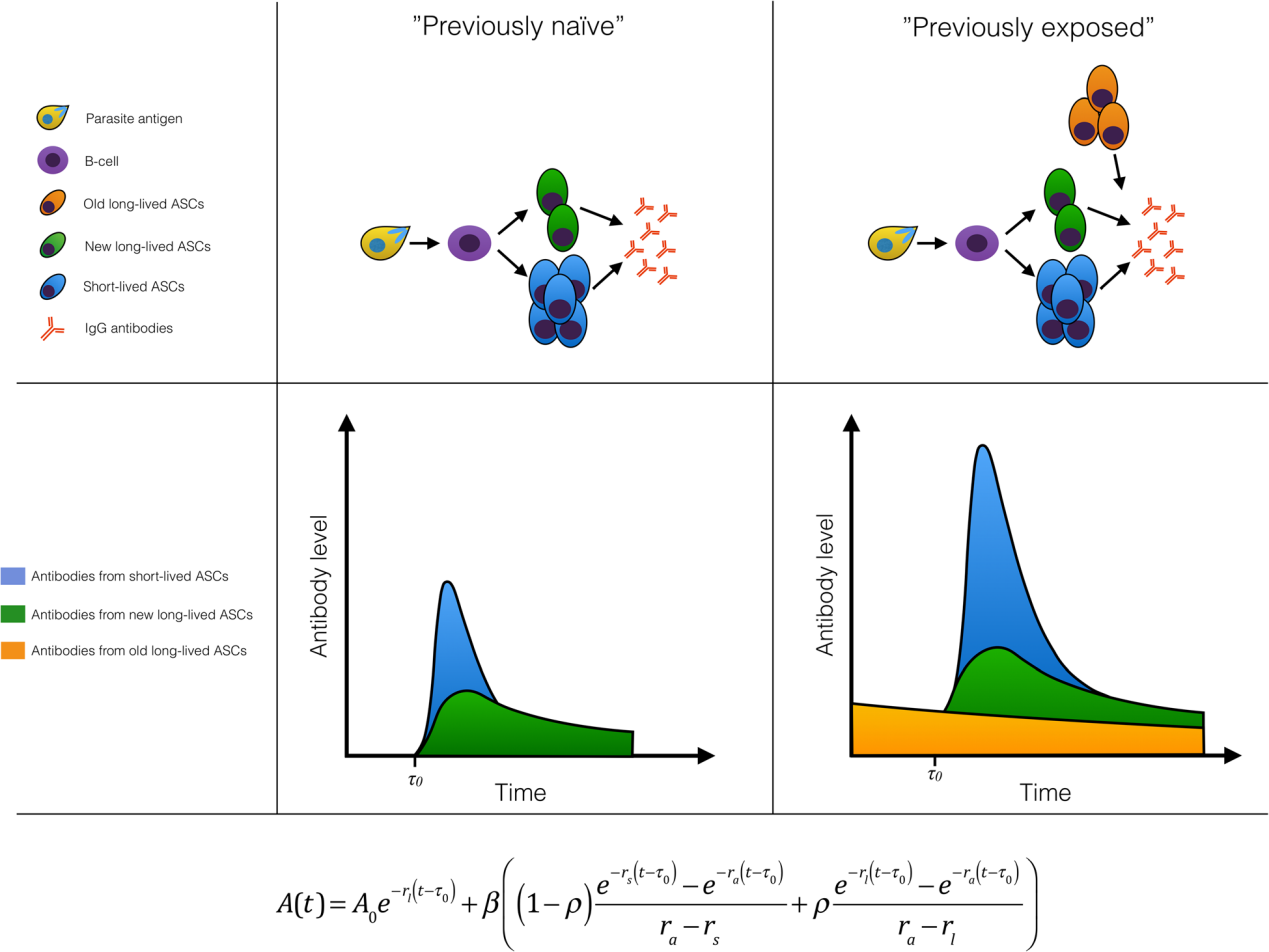
Schematic representation of the antibody kinetics model. The top row represents how the model captures the underlying immunological processes in both exposure groups, the middle row depicts the change in antibody levels over time (blue, antibodies generated by short-lived antibody-secreting cells (ASCs); green, antibodies generated by newly formed long-lived ASCs; orange, antibodies generated by previously established long-lived ASCs), and the bottom row displays the model equation. It is assumed that at time *τ*_0,_ prior to presentation to hospital, antigen exposure leads to the proliferation and differentiation of B cells generating an amount β of antibody-secreting cells (ASCs). A proportion of the ASCs (*ρ*) are long-lived (green) and decay at rate *r*_*l*_ while a proportion (1 − *ρ*) are short-lived (blue) and decay at rate *r*_*s*_. All ASCs produce antibodies that decay at rate *r*_*a*_. Previously exposed individuals, who have had prior *P*. *falciparum* infections, may maintain a level of antibodies (*A*_0_) generated by old long-lived ASCs (orange) from previous infections, which decay at rate *r*_*l*_ and produce antibodies that decay at rate *r*_*a*_. Previously naïve individuals, who suffer a primary *P*. *falciparum* infection, are assumed to have no pre-existing antibodies or ASCs at the onset of infection (*A*_0_ = 0)

We used a previously described mathematical model [5] to estimate the antigen- and subclass-specific boosting and decay of antibodies and ASCs. We developed an extension of the model to account for differences between individuals with or without prior malaria exposure. A schematic model representation is presented in Fig. 1. Briefly, the model assumes that in previously malaria-naïve individuals, the antigenic stimulus from the malaria infection leads to the proliferation and differentiation of B cells into both short- and long-lived ASCs that secrete IgG molecules, causing an initial rapid increase in antibody levels. Short- and long-lived ASCs decay at different rates leading to a bi-phasic decay in antibody levels over time. The model assumes that individuals with previous exposure may in addition have pre-existing slowly decaying long-lived ASCs generated during previous infections. The model estimates the half-life of secreted antibody molecules and both short- and long-lived ASCs and accounts for exposure-related differences in initial antibody levels, in the magnitude of boosting upon infection, and in the proportion of short- versus long-lived ASCs.

We assume that the infection causes antibody levels to rise *τ*_0_ days before the individual presents to the hospital (where *τ*_0_ is a parameter estimated for each individual) and that *A*(*t*) is the antibody level at time *t* > *τ*_0_ and is given by the following equation:

$$ A(t)={A}_0{e}^{-{r}_l\left(t-{\tau}_0\right)}+\beta\;\left(\left(1-\rho \right)\;\frac{e^{-{r}_s\;\left(t-{\tau}_0\right)}-{e}^{-{r}_a\;\left(t-{\tau}_0\right)}}{r_a-{r}_s}+\rho \frac{e^{-{r}_l\;\left(t-{\tau}_0\right)}-{e}^{-{r}_a\;\left(t-{\tau}_0\right)}}{r_a-{r}_l}\right) $$

where *r*_*a*_ is the rate of decay of IgG molecules; *r*_*s*_ and *r*_*l*_ are the rates of decay of short- and long-lived ASCs, respectively; *β* is the boost in ASCs following infection at time *τ*_0_; and *ρ* is the proportion of ASCs that are long-lived. *A*_0_ is the pre-existing levels of antibodies. For previously naïve participants *A*_0_ = 0. The models were fitted separately for *P*. *falciparum* antigen-specific total IgG and all subclasses (IgG_1–4_) in a Bayesian framework, and mixed-effects methods were used to capture the natural variation in antibody kinetics between individuals while estimating the average value and variance of the parameters across the entire cohort. To provide a validation of the estimated half-lives of the response to *P*. *falciparum* antigens, the model was also fitted to the data on total IgG levels to TTd, an unrelated antigen for which the half-life of the response is well characterised [14, 46]. A detailed description of the model fitting procedure is included as supplementary information (Additional file 1: Supplementary methods). R version 3.3.3 (The R Foundation for Statistical Computing, Vienna, Austria) was used for data management, statistical analysis, and mathematical modelling of antibody kinetics.

## Results

### Previous exposure and levels of antibodies to malaria antigens and tetanus toxoid

The “previously exposed” and the “previously naïve” individuals did not differ significantly with regard to age, sex, time from onset of symptoms to diagnosis, parasitaemia, or symptoms of severe malaria (Table 1). Antibody levels to *P*. *falciparum* antigens were positively correlated, in particular, for the two allelic variants of AMA-1 and MSP-3 (range *r* 0.93–0.98). However, antibody levels to *P*. *falciparum* antigens were not correlated with antibody levels to TTd (Additional file 1: Figure S1). At the time of diagnosis, previously exposed individuals had mounted significantly higher levels of total IgG to *P. falciparum* schizont extract, and all of the recombinant malaria antigens, than previously naïve individuals (Additional file 1: Figure S2). The difference was greatest for MSP-2_Dd2 (linear regression: fold difference 5.6, 95% CI 2.5–13.1, *p* < 0.001) while smallest for MSP-3_3D7 (linear regression: fold difference 2.0, 95% CI 1.1–3.6, *p* = 0.04). The individual tetanus vaccination status (i.e. number and timing of doses received) was not known, but all study participants were highly seroreactive towards TTd (Additional file 1: Figure S2). Geometric mean of *P*. *falciparum*-specific total IgG levels at diagnosis was neither associated with cumulative time of residency in a malaria-endemic area (years) for any exposure group (linear regression: estimates ranged from − 0.002 [95% CI − 0.0498–0.0467, *p* = 0.95] for MSP3_3D7 to 0.025 [95% CI − 0.047–0.097, *p* = 0.505] for MSP2_Dd2) nor with time since residency in a malaria-endemic area (years) within the previously exposed group (linear regression: estimates ranged from − 0.017 [95% CI − 0.048–0.015, *p* = 0.298] for RH5 to 0.010 [95% CI − 0.036–0.055, *p* = 0.683] for MSP2_CH150/9). Late treatment failures were observed in five previously naïve individuals (Table 1); however, the overall antibody boosting and decay patterns in these individuals did not differ significantly from those of the remaining participants in the previously naïve group.

**Table 1:** Descriptive statistics of the study participants

	Previously naïve	Previously exposed
Number of participants	20	41
Female sex (%)	3 (15.0)	7 (17.1)
Median age, years (range)	34 (21–59)	40 (27–70)
Median cumulative time of residency in an endemic area, years (range)	0 (0–3)	25 (13–39)
Median time since residency in an endemic area, years (range)	–	14 (0–46)
Median time from symptom onset to diagnosis, days (range)	3 (0–12)	3 (1–13)
Median parasitaemia, % infected RBCs (range)	0.9 (< 0.1–8.0)	0.3 (< 0.1–17)
Late treatment failure* (%)	5 (25)	0 (0)
Severe malaria^†^ (%)	1 (5)	4 (9.7)
Treated in intensive care unit (%)	1 (5)	2 (4.9)
Initial intraveneous artesunate treatment (%)	6 (30)	10 (24.4)

*Presented with recrudescent parasitaemia and fever 20–28 days following initial treatment

^†^Severe malaria was defined according to the WHO classification

### Modelling antibody boosting and decay patterns

**Fig. 2:**
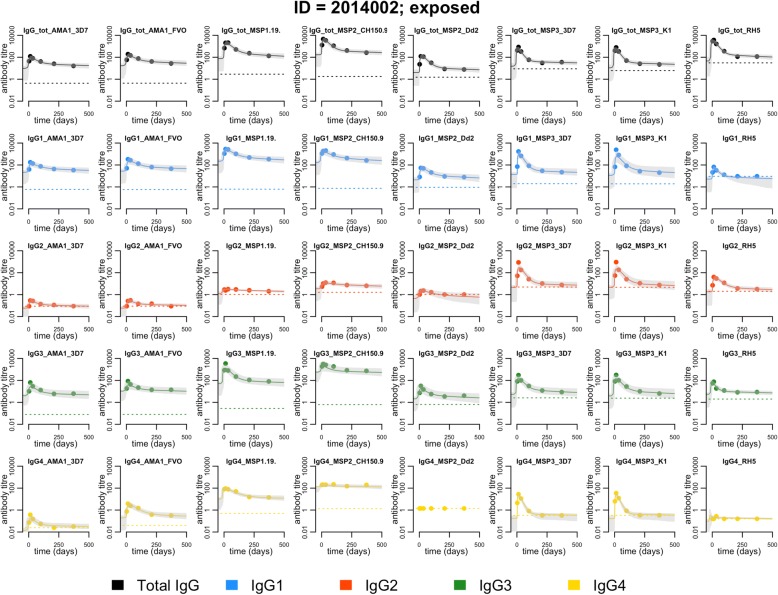
Antibody kinetics for a representative study subject with previous exposure to *P*. *falciparum* (ID: 2014002). Each panel represents one antigen and either total IgG or one IgG subclass. The dots denote the individual sample antibody level in arbitrary units. The solid line denotes the model predicted antibody boost and decay patterns relative to the collection of the first sample at time *t* = 0 and the shaded grey area the 95% credible interval of the prediction. The dotted line represents the assay lower limit of quantification

Data and model fits for antigen-specific total IgG and IgG subclasses are presented for a representative study participant in Fig. 2 and for additional study participants as supplementary information (Additional file 1: Figure S3-S5). Boosting and decay patterns were consistent across antigens for both total IgG and all IgG subclasses. As illustrated in Fig. 2, antigen-specific IgG_2_ and IgG_4_ levels were often low or even undetectable and this slightly complicated the fitting of the model to data for these subclasses. However, when exceeding the lower limit of quantification, IgG_2_ and IgG_4_ antibody levels displayed boosting and decay patterns consistent with what we observed for IgG_1_ and IgG_3_, lending support to our efforts to estimate the antigen-specific antibody decay rate for all IgG subclasses. The model-estimated population-level parameters and corresponding variance parameters are presented as supplementary information (Additional file 2: Table S1).

#### Antibody kinetics of total IgG responses

**Fig. 3:**
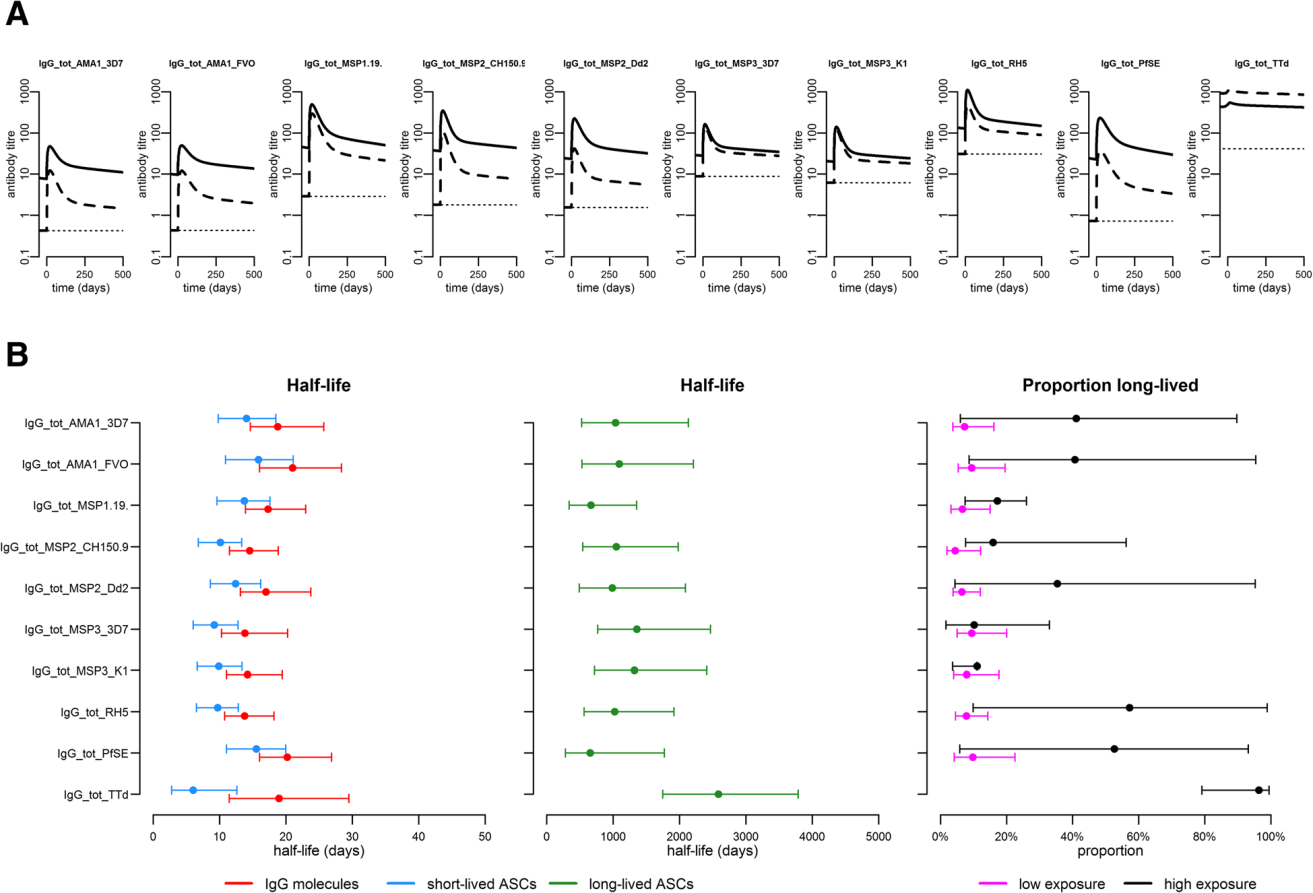
Model-predicted antibody kinetics during follow-up in individuals with or without previous malaria exposure. **a** The geometric mean antigen-specific total IgG levels over time for malaria antigens and TTd in each exposure group relative to the collection of the first sample at time *t* = 0. Solid lines denote the previously exposed while dashed lines denote the previously malaria naïve. The antibody levels in arbitrary antibody units are directly comparable only within the antigen. The dotted line represents the assay lower limit of quantification. **b** Parameter estimates for the kinetics of the total IgG response to *P*. *falciparum* antigens and TTd. Dots denote the model parameter estimates and capped error bars the corresponding 95% CrI. The different parameters are indicated by colours: half-life of antibody molecules (red), half-life of short-lived ASCs (blue), half-life of long-lived ASCs (green), and the proportion of long-lived ASCs in previously naïve (magenta) and in the previously exposed (black). Individuals were not stratified by prior malaria exposure status when the model was fitted to data on the response to TTd. The estimated population-level parameters with corresponding variance parameters are also available in Additional file 2: Table S1

Geometric mean malaria-specific IgG levels initially increased rapidly in both exposure groups (Fig. 3a). The estimated peak in antibody levels occurred around 14 days after diagnosis, after which antibody levels initially declined rapidly followed by a second phase of a slower decay during the remainder of the one-year follow-up (Fig. 3a). Geometric mean IgG levels were consistently higher in previously exposed individuals for all malaria antigens except MSP-3 (Fig. 3a). Differences between the groups were particularly evident in the greater boost size (Additional file 2: Table S1) and in the maintenance of higher plateau antibody levels at the end of follow-up (Additional file 3: Table S2). Depending on the antigen, the previously exposed individuals on average maintained 1.7–8.8-fold greater antibody levels than previously naïve individuals at the end of follow-up. The relative difference was greatest for schizont extract and AMA-1 antigens while smallest for RH5 (Fig. 3a, Additional file 3: Table S2). For RH5, the complementary ELISA analysis confirmed that the overall magnitude of the response was consistent with the previous observations from natural malaria exposure (median, < 0.5 μg/mL; range, < 0.5–3.3 μg/mL) [45].

#### Half-life of total IgG antibody responses to *P*. *falciparum* schizont extract, recombinant antigens, and tetanus toxoid

The half-life of circulating IgG molecules varied slightly with antigen specificity and ranged from 14 to 21 days (Fig. 3b). The half-life of short-lived ASCs was shorter and ranged from 10 to 16 days. It was shortest for ASCs producing antibodies specific for MSP-3 antigens while longest for ASCs producing antibodies to AMA-1, but credible intervals (CrI) were largely overlapping (Fig. 3b). The estimated half-life of long-lived ASCs, based on malaria-specific total IgG, ranged from 655 to 1357 days (i.e.1.8–3.7 years) depending on the antigen and, although CrIs were overlapping, tended to be shorter for schizont extract and MSP-1_19_ while longer for MSP-3 (Fig. 3b). For IgG to TTd, the half-lives of secreted IgG molecules and short-lived ASCs (estimated by the model by design) were 19 and 6 days, respectively (Fig. 3b). The half-life of long-lived ASCs producing antibodies to TTd was 2585 days, i.e. 7.1 years (95% CrI 5–11.4 years), and thus considerably longer than that of long-lived malaria-specific ASCs.

#### Relative contribution of short- versus long-lived ASCs to the overall antigen-specific total IgG response

The relative contribution of long-lived ASCs to the overall antigen-specific IgG response varied with antigen but was larger in previously exposed individuals (Fig. 3b). In previously naïve individuals, only a small proportion of the ASCs generated in response to infection, 4–10% depending on the antigen, were estimated to be long-lived. However, in previously exposed individuals, there was a substantial contribution from long-lived ASCs, generated both during the present and past infections, ranging from 10 to 57% depending on the antigen tested (Fig. 3b). For TTd, the proportion of long-lived ASCs was estimated to be 96% across both exposure groups (Fig. 3b), consistent with a non-boosted antibody response maintained entirely by long-lived ASCs.

#### In-depth characterisation of the kinetics and longevity of the antigen-specific IgG subclass response to eight recombinant *P*. *falciparum* antigens

**Fig. 4:**
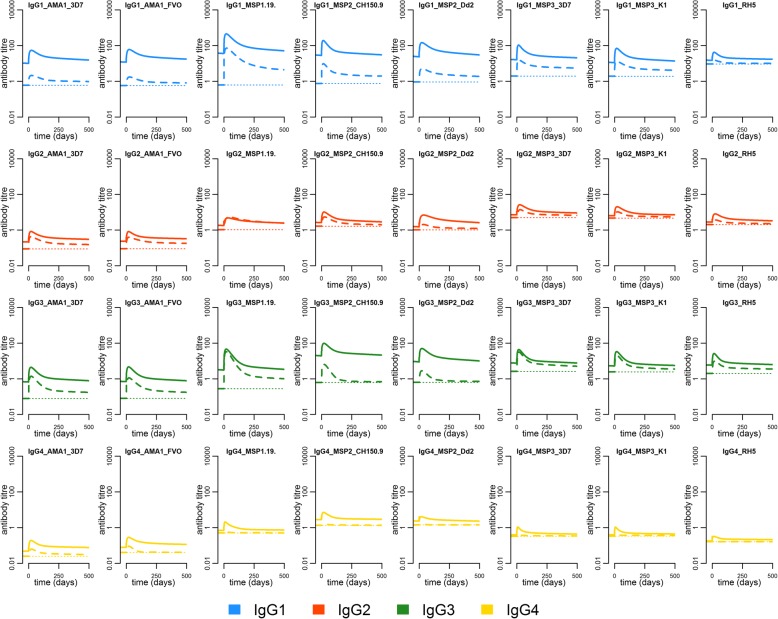
Geometric mean kinetics of the *P*. *falciparum*-specific IgG subclass response. The geometric mean antibody kinetics over time in each of the exposure groups relative to the collection of the first sample at time *t* = 0. Each panel represents the IgG subclass response of a particular antigen specificity. Blue, red, green, and yellow denote the IgG_1_, IgG_2_, IgG_3_, and IgG_4_ response, respectively. Solid lines represent the previously exposed and the dashed lines the previously naïve. The antibody levels in arbitrary antibody units are directly comparable only within the antigen and IgG subclass. The dotted line represents the assay lower limit of quantification. The estimated population-level parameters with corresponding variance parameters are available in Additional file 2: Table S1

The kinetics of each of the IgG subclass responses were similar to the overall kinetics of the total IgG response with the same antigen specificity (Fig. 4). Antibody levels were consistently higher in the previously exposed group for all antigen-specific IgG subclass responses, except the IgG_2_ response to MSP-1_19_ where levels and kinetics were similar between the groups. The difference was particularly evident in IgG_1_ to AMA-1 and IgG_3_ to MSP-2, where previously exposed individuals at the end of follow-up maintained on average 15.5–21.5-fold and 14.1–31.1-fold greater levels, respectively (Fig. 4, Additional file 3: Table S2). In addition, a difference was also clearly discernible for MSP-1_19_ and MSP-3, for which a difference was not evident in levels of total IgG (Fig. 4). On average, only the previously exposed individuals mounted a detectable IgG_4_ response and although levels were low, the difference in geometric mean IgG_4_ levels between exposure groups was substantial across all antigen specificities (Fig. 4).

In particular, for IgG_1_ and IgG_3_, the estimates of the half-lives of antibody molecules, and the short- and long-lived ASCs producing them, were similar to the corresponding estimates based on data for total IgG of the same antigen specificity. For IgG_1_ and IgG_3_ responses, half-lives ranged from 15 to 22 days, 9 to 16 days, and 800 to 1424 days (i.e. 2.1–3.9 years) for the antibody molecules, short-lived ASCs, and long-lived ASCs, respectively (Additional file 1: Figure S6). There were greater variability and uncertainty in estimates for IgG_2_ and IgG_4_ responses, and depending on the antigen specificity, half-lives ranged from 15 to 27 days, 4 to 20 days, and 729 to 2232 days (i.e. 2.0–6.1 years) for antibody molecules, short-lived ASCs, and long-lived ASCs, respectively (Additional file 1: Figure S6).

The previously exposed individuals were estimated to have a greater contribution from long-lived ASCs for most subclass responses (Additional file 1: Figure S6, Additional file 2: Table S1). In previously naïve individuals, the estimated proportion of long-lived ASCs was similar across antigen specificities within each IgG subclass and ranged from 2% (95% CrI 0–14) to 23% (95% CrI 9–42). In the previously exposed individuals, there was substantial variability between subclasses and antigen specificities, and the estimated proportion of long-lived ASCs ranged from 5% (95% CrI 2–89) to 85% (95% CrI 56–99) (Additional file 1: Figure S6).

#### Breadth of the total IgG and IgG subclass response over time

**Fig. 5:**
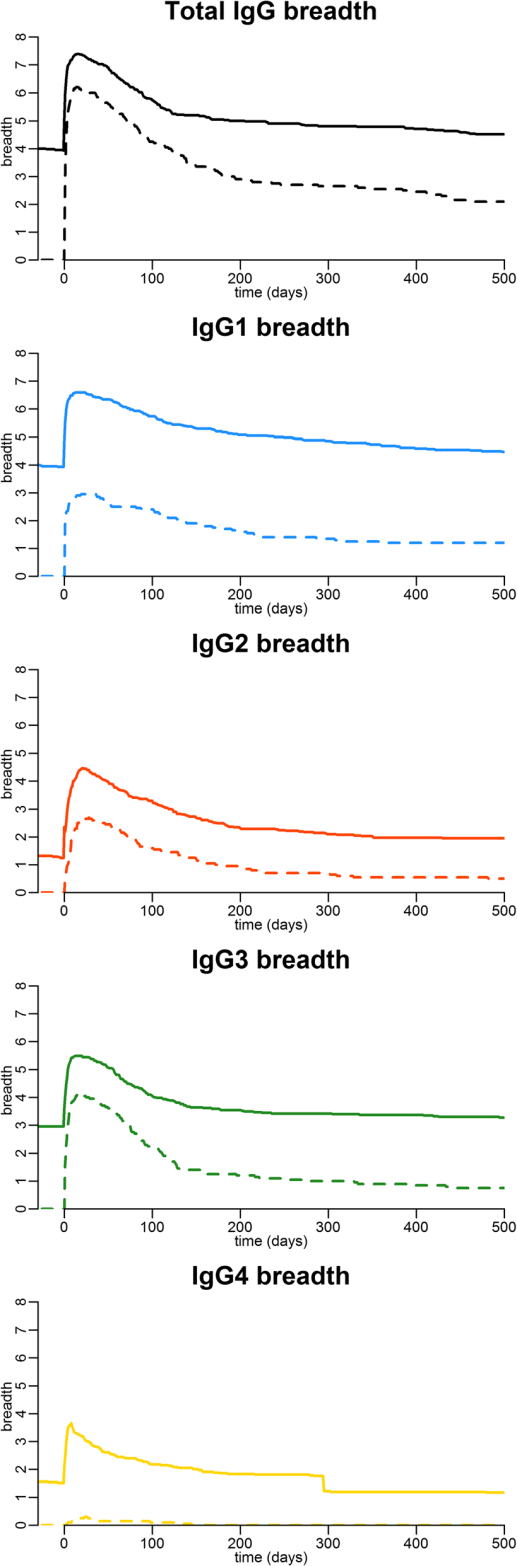
The average breadth of the antibody response for total IgG and IgG subclasses over time. Each panel represents either total IgG or one of the IgG subclasses. Black, blue, red, green, and yellow denote the total IgG, IgG_1_, IgG_2_, IgG_3_, and IgG_4_ response, respectively. The solid line and dashed lines represent the temporal kinetics in the average antibody breadth in previously exposed and previously naïve individuals, respectively, relative to the collection of the first sample at time *t* = 0. Antibody breadth is the number of recombinant *P*. *falciparum* antigens to which the individual displays antibody reactivity above a threshold of seropositivity (defined as the mean + 3 SD of the reactivity of 20 malaria unexposed controls)

The mean breadth of the total IgG and subclass responses (defined as the number of antigens to which an individual displayed seroreactivity above the threshold of seropositivity) reached its maximum within a month following malaria diagnosis and was greater among individuals with previous exposure (Fig. 5). Following the peak, it declined more slowly in previously exposed individuals, who also maintained a significantly higher antibody breadth at the end of follow-up. The patterns for the individual subclasses were consistent with those of total IgG, but the subclass-specific breadth was estimated to be slightly lower (Fig. 5). The difference in breadth, due to prior exposure, was greatest for the IgG_4_ response where previously naïve individuals rarely were able to mount an antibody response above the threshold of seropositivity. Individuals with previous exposure were, for most antigens, estimated to remain seropositive substantially longer. The antigen-specific half-life of seropositivity (i.e. the number of years it takes for half of the individuals to become seronegative) in the previously exposed group ranged from 0.11 to 19.92 years compared to 0.03 to 5.67 years in the previously naïve (Additional file 4: Table S3).

## Discussion

We investigated the kinetics of the antibody response after a naturally acquired *P*. *falciparum* infection by studying a cohort of differentially exposed travellers followed for 1 year after treatment in complete absence of re-exposure. We observed greater magnitude, breadth, and longevity of the response to all tested *P*. *falciparum* antigens (AMA-1, MSP-1_19_, MSP-2, MSP-3, and RH5) in individuals with previous repeated malaria exposure. Through mathematical modelling of longitudinal data on antigen-specific total IgG and IgG subclass responses, we provided quantitative estimates of the kinetics of antibodies and short- and long-lived ASCs. The half-lives of the antimalarial long-lived ASCs were notably short in comparison with tetanus-specific long-lived ASCs, and although long-lived ASCs were acquired following primary malaria infection, they represented only a small fraction of the total ASCs generated. We observed a greater longevity of the antibody response in previously exposed individuals and show that this can be explained by the maintenance of a greater number of long-lived ASCs.

During the year following infection, geometric mean antimalarial antibody levels of all antigen specificities (total IgG and all IgG subclasses) were consistently higher in individuals with prior malaria exposure. The kinetics of the antigen-specific total IgG response, which is reported to consist predominantly of the IgG_1_ and IgG_3_ subclasses [47–50], was also best reflected in the subclass kinetics of either IgG_1_ or IgG_3_. High-level IgG_1_ and IgG_3_ responses to several merozoite surface antigens have been associated with clinical protection [47–50], and here, previously exposed, but not previously naïve, individuals mounted high IgG_1_ and IgG_3_ responses to AMA-1 and MSP-2. Furthermore, a large proportion of individuals with previous exposure, but almost none of the previously naïve, mounted detectable IgG_4_ responses irrespective of antigen specificity. The role of IgG_4_ in malaria is to date incompletely resolved but increased levels have been associated with prolonged or chronic antigen exposure [24, 48, 51].

Although the half-lives of antibodies and ASCs have previously been estimated from longitudinal antibody data in children living in endemic areas [5], no quantitative estimates are previously available for primary infections or from cohorts followed in complete absence of re-exposure. Here, the estimated half-lives of *P*. *falciparum*-specific short-lived ASCs ranged from a few days to a few weeks whereas the half-life of long-lived ASCs range from approximately 2 to 6 years depending on IgG subclass and antigen specificity (2 to 4 years for total IgG, IgG_1_, and IgG_3_ versus 2 to 6 years for IgG_2_ and IgG_4_). There was a tendency for the estimated half-life of long-lived IgG_2_ and IgG_4_ secreting ASCs to be longer than for IgG_1_, IgG_3_, and total IgG. However, this could be due to biases related to the small number of samples with seroreactivity exceeding the lower limit of detection of the assay, particularly among the previously naïve. The estimated decay rates of antibody molecules as well as both short- and long-lived ASCs are highly consistent with previous estimates for antibodies and ASCs specific for MSP-1, MSP-2, and AMA-1 in African children [5, 19, 20].

The greater boosting of antibody levels in previously exposed individuals is indicative of a greater number of short-lived ASCs, likely due to the presence of a memory B cell response that upon reinfection rapidly proliferates and differentiates into short-lived ASCs [52–54]. It has previously been demonstrated that malaria-specific memory B cells can be acquired already after a few infections and that they may be maintained, and detected in the peripheral blood, for several years in absence of re-exposure [55–57]. Furthermore, it has been demonstrated that memory B cells can be maintained independently of circulating antibodies and vice versa [13, 56, 58, 59].

Interestingly, the previously naïve individuals were found to acquire a long-lived component of the antibody response during this primary *P*. *falciparum* infection. However, this component was small, and in previously naïve individuals, only minor proportions of the ASCs generated in response to the infection were estimated to be long-lived (e.g. 4–10% for total IgG of all antigen specificities). Individuals with prior exposure were estimated to maintain a greater proportion of long-lived ASCs (e.g. 10–56% for total IgG), supported also by the maintenance of two- to ninefold higher antibody levels at the end of follow-up approximately a year after the acute infection. Because of the rapid decay in short-lived ASCs, the antibodies they produce will disappear completely from the circulation within a few months after the infection has been cleared. Any circulating antibodies remaining at the end of follow-up will be maintained entirely by long-lived ASCs. The magnitude of the antibody response at the end of the follow-up will therefore be proportional to the number of long-lived ASC [22, 23]. The higher levels of antibodies observed in previously exposed individuals compared to previously naïve individuals thus reflect the maintenance of a greater absolute number of long-lived ASCs. The greater longevity of the response in previously exposed individuals is further reflected in the greater antibody breadth (i.e. the number of antigens to which an individual responds), which has been previously associated both with protection from disease and with time since the last infection [36, 60, 61].

The mathematical model used represents necessary simplifications of the underlying processes that regulate the generation and survival of ASCs [5]. Differences between previously naïve and exposed individuals are assumed to be due to the differences in pre-infection antibody levels, differences in antibody boosting, and differences in the proportions of short- versus long-lived ASCs [5, 52–54]. In our framework, differences in the longevity of the response could be modelled by varying either the proportion of ASCs that are long-lived or the half-life of the long-lived ASCs or by varying both. However, due to the relatively small sample size, it is not possible to significantly test for differences between the two groups while allowing both of the above to vary. We therefore make the assumption that the half-lives of ASCs are the same in both groups but that they differ in terms of the proportion of long-lived ASCs. Despite this simplification, the current model provides a good fit to the data on antimalarial antibody responses. Furthermore, when fitted to data on IgG antibody responses to TTd, in the same individuals, it reproduces previously published estimates of the half-life of the antibody response to tetanus toxoid of approximately 7–14 years [14, 46, 62]. This provides a validation of the current model structure and the reliability of the model estimates for the kinetics of the antibody response to malaria antigens.

Because the exposure-dependent differences in antibody kinetics can be described by the differences in the size of the antibody boost and the acquired proportion of long-lived ASCs, we suggest that a more long-lived antibody response to malaria is acquired slowly over time by small consecutive additions to the pool of long-lived ASCs with each repeated infection. The relatively small number of long-lived ASCs generated by each new infection, as indicated by the low proportion of long-lived ASCs in the previously naïve group, contributes to the slow acquisition of a long-lived high titre antibody responses. This is supported by the data from Ghana and the Gambia, where the proportion of long-lived ASCs in children was estimated to increase with age [5, 19], and could also partly explain the observation of a more rapid seroreversion in children, compared to adults, in endemic areas following interruption of transmission [63].

To date, most successful vaccines induce protection mediated through antibodies that are maintained following just a few immunisations [4]. The most advanced malaria vaccine, RTS,S, provides only partial protection, and vaccine efficacy appears to wane quickly as antibody responses decay [64]. All of the antigens tested in the present study are either current or previous vaccine candidates with clinical trials ongoing for AMA-1 and RH5 [45, 65]. Most of them, except RH5, are highly polymorphic and have been selected as candidate vaccines partly because of their immunogenicity in the context of natural malaria infection [4, 66, 67]. However, here we find that despite the high immunogenicity, antibody responses decay rapidly in the absence of re-exposure. It is possible that the high rate of decay together with the highly polymorphic nature of many malaria antigens contributes significantly to the slow acquisition of clinical immunity [16, 68, 69]. Furthermore, despite an enhanced expansion of parasite-specific antibodies in previously exposed individuals, they did not exhibit clinical protection against malaria disease, suggesting that B cells archived within the memory compartment either have non-protective specificities or that their reactivation is too late to control the acute infection [16, 68]. RH5, in contrast, is a relatively conserved protein. It is not one of the major targets of naturally acquired antibodies [70] but a leading blood-stage vaccine candidate that has shown promising results in vitro as well as in non-human primate models [71, 72]. Nonetheless, the estimated longevity of the response to RH5 was similar to that of the highly immunogenic and much more polymorphic antigens tested, all of which were short in comparison with the response to the well-studied tetanus toxoid vaccine antigen. Accumulating evidence suggests that the immune environment induced by the malaria parasite inhibits the development of a long-lived antibody response. Development of both long-lived ASCs and immune memory are hampered by a dysregulation of the B cell response, where impaired T cell help and germinal centre formation [73, 74] lead to preferential induction of short-lived ASCs and the generation of the so-called atypical memory B cells [5, 55, 60, 75, 76]. The decay patterns of malaria-specific antibody responses observed here following natural infection are similar to those observed in African children following vaccination with the RTS,S vaccine [64]. These findings emphasise the importance of tailoring a malaria vaccine to skew the humoral immune response towards the generation of long-lived ASCs by improving delivery platforms and adjuvants and by optimising dosage and vaccine regimens [67].

Serology has proven to be a promising tool to monitor medium- and long-term trends in malaria transmission intensity, particularly in low transmission settings [6]. Available tools have largely been based on the data from cross-sectional epidemiological surveys [6, 8], but a challenge when modelling cross-sectional serological data is the issue of parameter identifiability [10]. For example, when data are limited, it may be impossible to distinguish between the scenario in which individuals acquire and lose antibodies rapidly or the opposite in which antibodies are both acquired and lost slowly leading to spurious estimates of transmission intensity [10]. Improvement of current serological tools for transmission monitoring requires reliable quantitative estimates of the underlying parameters, e.g. the antigen-specific antibody decay rate, as well as the identification of novel serological markers, or combinations of markers, that discriminate between recent or more distant exposure [7, 61]. In the present context, the difference in antibody kinetics between the exposure groups was greatest for AMA-1 and MSP-2 antigens and these antigens appeared most informative to differentiate individuals based on the prior exposure. However, additional serological markers with shorter half-lives would be needed to accurately predict the timing of the last infection. We suggest that the longitudinal study of differentially exposed travellers may serve as a useful model system to identify such candidate serological markers that can subsequently be validated under field conditions.

## Conclusions

This prospective study of differentially exposed travellers treated for *P*. *falciparum* infection gives new insight into the acquisition and maintenance of the antibody response to eight malaria vaccine candidate antigens. Through mathematical modelling of longitudinal antibody data, using an approach that is widely applicable for the study of the humoral response to both infection and vaccination, we provide quantitative estimates of the exposure-dependent kinetics of antibodies and ASCs. The data suggest that the overall short-lived nature of the antibody response to natural infection can be attributed to a combination of a short half-life and an inefficient acquisition of long-lived antimalarial ASCs. The quantitative estimates of the total and IgG subclass specific kinetics of the response may help guide strategies for further vaccine development and improve serological tools for malaria transmission surveillance.

## Additional files

Additional file 1: Supplementary information. Supplementary methods including technical details of antibody assays and model fitting procedures. **Figure S1**-**S6.** (PDF 7872 kb)

Additional file 2: **Table S1.** Antibody kinetics model estimates of the population level parameters with corresponding variance parameters. (XLSX 39 kb)

Additional file 3: **Table S2.** Antibody kinetics model estimates of the geometric mean antibody levels at the end of follow-up. (XLSX 27 kb)

Additional file 4: **Table S3.** Antibody kinetics model estimates of the time-to-seroreversion. (XLSX 22 kb)

## Abbreviations

AMA-1: Apical membrane antigen 1
ASC: Antibody-secreting cell
CHMI: Controlled human malaria infection
CrI: Credible interval
IgG: Immunoglobulin G
MFI: Median fluorescent intensity
MSP: Merozoite surface protein
OD: Optical density
PfSE: Plasmodium falciparum schizont extract
RH5: Reticulocyte-binding protein homologue 5
TTd: Tetanus toxoid
