# Supplementary material for: Antibody responses to merozoite antigens after natural Plasmodium falciparum infection: kinetics and longevity in absence of re-exposure
Source: BMC Med. 2019 Jan 30;17:22. doi: 10.1186/s12916-019-1255-3 (PMC6352425; doi:10.1186/s12916-019-1255-3)
Supplement: Supplementary file 1 — Supplementary information. Supplementary methods including technical details of antibody assays and model fitting procedures. Figure S1-S6. (PDF 7872 kb) [file 12916_2019_1255_MOESM1_ESM.pdf]

Additional file 1

Supplementary Information for:

Antibody responses to merozoite antigens after natural *Plasmodium falciparum* infection: kinetics and longevity in absence of re-exposure

Yman V, White MT, Asghar M, Sundling C, Sondén K, Draper SJ, Osier FHA, Färnert A

Supplementary methods: Antibody assays

An ELISA was used to quantify total IgG levels to shizont extract (3D7 clone) according to a previously described protocol . Multiplex bead based immunoassays were used to quantify IgG antibody responses, total IgG and subclasses $IgG_{1\text{-}4}$, to recombinant *P. falciparum*. Antigens included the 19 kDa fragment of MSP-1 (MSP-1_{19}), full-length RH5 (based on the 3D7 clone sequence), two allelic variants of each of MSP-2 (CH150/9 and Dd2), MSP-3 (3D7 and K1) and AMA-1 (3D7 and FVO). The assay was performed separately for detection of total IgG and each of the four IgG subclasses, $IgG_{1\text{-}4}$. A singleplex bead based immunoassay was used to quantify total IgG antibodies to TTd.

Each antigen was coupled to a spectrally unique set of paramagnetic beads (Bio-Rad Laboratories, Hercules, CA, USA), using the Bio-Plex amine coupling kit (Bio-Rad Laboratories) at an antigen concentration of 2.5 µg per million beads for malaria antigens and 5 µg per million beads for TTd. For the malaria multiplex assay, all antigen-coupled bead sets were mixed and 2500 beads per antigen were distributed into each well of a Bio-Plex Pro Flat Bottom plate (Bio-Rad Laboratories). Samples were diluted in phosphate buffered saline (PBS) and Tween 20 with 1 % bovine serum albumin (BSA) and analysed at a dilution of 1:1000 for total IgG, 1:500 for IgG_1 and IgG_3, 1:300 for IgG_2, and 1:100 for IgG_4. Samples were incubated for 1 hour at room temperature with continuous shaking. After washing four times with PBS-Tween, 50 µl of R-phycoerythrin (PE) conjugated secondary antibody diluted in PBS-Tween with 1% BSA was added and incubated for 30 minutes at room temperature. $F(ab')_2$, goat anti-human IgG FC-R-PE (1 mg/mL, Jackson ImmunoResearch Laboratories Inc., West Grove, PA, USA) in 1:300 dilution, mouse anti-human IgG_1 hinge-PE (0.1 mg/mL, clone 4E3, Southern Biotech, Birmingham, AL, USA), mouse anti-human IgG_2 Fc-PE (0.1 mg/mL, clone HP6002, Southern Biotech), mouse anti-human IgG_3 hinge-PE (0.1mg/mL, clone HP6050, Southern Biotech) and mouse anti-human IgG_4 Fc-PE (0.1 mg/mL, clone HP6025, Southern Biotech), all in 1:100 dilution, were used to detect total IgG and $IgG_{1\text{-}4}$, respectively. Beads were then washed and resuspended in 100 µl of PBS-Tween with 1% BSA and results were analysed on a Bio-Plex 200^{TM} instrument (Bio-Rad Laboratories). The same procedure as outlined above for total IgG was used for the singleplex assay for total IgG to TTd.

Positive and negative controls (pooled plasma from adult highly malaria exposed Kenyan donors and malaria unexposed Swedish donors, respectively) and a standard calibrator of serially diluted purified IgG from malaria immune donors were run on each plate in the malaria antigen multiplex assay. In the TTd assay the positive control consisted of the same highly reactive plasma pool from Kenyan donors. A serially diluted "in-house" reference sera from highly TTd reactive volunteers that had been recently booster immunised using diTeBooster® (AJ Vaccines, Denmark) was used as a standard calibrator.

For each antigen and each IgG subclass, the assay optical density (OD) or median fluorescent intensity (MFI) was converted to a relative concentration in arbitrary units (AU/mL) by interpolation from the standard calibrator curve using a five-parameter sigmoidal curve fitting. The highest concentration standard calibrator in each assay was assigned an arbitrary value of 960 AU/mL irrespective of its signal intensity. The interpolated concentration values were scaled by multiplying by the relative sample dilution factor (e.g. 10 for total IgG, 5 for IgG_1 and IgG_3, 3 for IgG_2, and 1 for IgG_4). The arbitrary antibody units are directly comparable only within each assay i.e. within antigen and IgG subclass. The assay lower limit of quantification (LLoQ) was established as the lowest concentration standard calibrator sample that displayed highly repeatable signal intensity measurements above the background noise across all experiments (defined as a CV% of all replicates of less than 20%). All samples and controls were assayed in duplicate and sample replicates with reactivity within the measuring range of the assay and a coefficient of variation (CV%) of above 20% were repeated. Samples with reactivity above the assay range were repeated at a higher dilution. A threshold of seropositivity was defined as the mean reactivity of 20 malaria unexposed controls plus three standard deviations.

Supplementary methods: Mathematical model and statistical methods

Antibody kinetics model

Following first exposure to blood-stage *P. falciparum* infection, we assume that the proliferation and differentiation of B cells leads to a boost in antibody secreting cells (ASC) of size β. A proportion ρ of these ASCs are assumed to be long-lived with half-life d_l, with a proportion 1 – ρ being short-lived with half-life d_s. It is assumed that all ASCs secrete IgG molecules which decay with a half-life d_a. The antibody level of an individual at time *t* after their first exposure to malaria at time τ_0 is given by:

$$A(t)=\beta\left((1-\rho)\frac{e^{-r_s(t-\tau_0)}-e^{-r_a(t-\tau_0)}}{r_a-r_s}+\rho\frac{e^{-r_l(t-\tau_0)}-e^{-r_a(t-\tau_0)}}{r_a-r_l}\right)$$

where r_a is the rate of decay of IgG molecules, related to the half-life of IgG molecules according to $r_a=\frac{\log(2)}{d_a}$.

r_s and r_l are similarly defined.

Individuals with a past history of exposure to *P. falciparum* will have some pre-existing level of antibodies (A_0) at the time of infection (τ_0). The rate of decay of this pre-existing antibody response is determined by the rate of decay of long-lived ASCs r_l. The antibody level of an individual with previous exposure at time *t* after their most recent exposure to malaria at time τ_0 is given by:

$$A(t) = A_0 e^{-r_l(t-\tau_0)} + \beta\left((1-\rho)\frac{e^{-r_s(t-\tau_0)} - e^{-r_a(t-\tau_0)}}{r_a - r_s} + \rho\frac{e^{-r_l(t-\tau_0)} - e^{-r_a(t-\tau_0)}}{r_a - r_l} \right)$$

A schematic overview of the model is shown in Figure 1 (main text).

Fitting the model to data

The model was fitted to longitudinal antibody level measurements from all participants. Mixed effects methods were used to capture the natural variation in antibody kinetics between individual participants, whilst estimating the average value and variance of the immune parameters across the entire cohort of individuals. The models were fitted in a Bayesian framework using Markov Chain Monte Carlo (MCMC) methods. Mixed effects methods allow local parameters to be estimated for each participant individually, with these local (or mixed effects) parameters being drawn from global distributions. For example, for each participant *n* the half-life of the short-lived ASCs may be estimated as d_s^n (an individual specific parameter). These *N* estimates of the local parameters d_s^n will be drawn from a probability distribution. A log-Normal distribution is suitable as it has positive support on $[0, \infty)$. Thus we have $\log(d_s^n) : N(\mu_s, \sigma_s^2)$. The mean d_s and the variance Σ_s^2 of the estimates of d_s^n are given by $d_s = e^{\mu_s + \frac{\sigma_s^2}{2}}$ and $\Sigma_s^2 = \left(e^{\sigma_s^2} - 1\right)e^{2\mu_s + \sigma_s^2}$. The relationship between the global parameters describing the population level distribution and the local parameters for each individual in the population are depicted in the schematic diagram below.

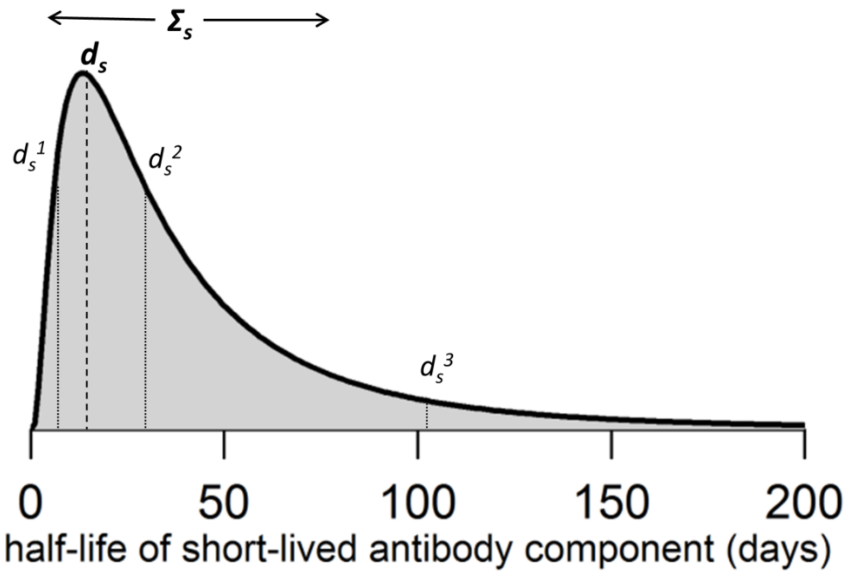

Schematic representation of the relationship between the global and local parameters for the half-life of the short-lived ASCs. It is assumed that half-lives are log-Normally distributed throughout the population. The global parameters define the mean d_s and standard deviation Σ_s of this distribution. The local parameters d_s^n for each of the n participants follow the log-Normal distribution defined by the global parameters. Three representative local parameters are shown for illustration.

Model likelihood

For participant *n* we have data on observed antibody levels $\mathrm{A}^n = \{a_1, ..., a_J\}$ at times $T^n = \{t_1, ..., t_J\}$. We denote $D^n = (A^n, T^n)$ to be the vector of data for participant *n*. For participant *n*, the six parameters A_0^n, β^n, d_s^n, d_l^n, d_a^n and ρ^n are estimated. Note that for participants in the low exposure group we have $A_0^n = 0$. These parameters are denoted $\theta^n = (A_0^n, \beta^n, d_s^n, d_l^n, d_l^n, \rho^n)$. The model predicted antibody levels will be $\{A(t_1), A(t_2), ..., A(t_J)\}$. We assume log-Normally distributed measurement error such that the difference between $\log(a_j)$ and $\log(\mathrm{A}(t_j))$ is Normally distributed with variance σ_{obs}^2. For model predicted antibody levels $\mathrm{A}(t_j)$ the data likelihood for participant *n* is given by

$$L_{\mathrm{mod}}^n\left(\theta^n \middle| D^n\right) = \prod_{j \in J} \frac{e^{-\frac{\left(\log(a_j) - \log(A(t_j))\right)^2}{2\sigma_{\mathrm{obs}}^2}}}{a_j \sigma_{\mathrm{obs}} \sqrt{2\pi}}$$

Mixed effects likelihood

As described above, for each individual there are up to 6 parameters to be estimated: $\theta^n = \left(A_0^n, \beta^n, d_s^n, d_l^n, d_l^n, \rho^n\right)$.

Individuals can be either from the 'previously unexposed' or 'previously exposed' groups. We assume that for individuals from both groups, the parameters d_s^n, d_l^n and d_a^n are drawn from the same population-level distribution. That is we assume that the average duration of short-lived and long-lived ASCs and IgG molecules is the same in both groups. We assume that the two exposure groups differ in terms of the magnitude of boosting to ASCs (β_{unexp} versus β_{exp}) and the proportion of ASCs that are long-lived (ρ_{unexp} versus ρ_{exp}). Thus for an individual in the previously unexposed group, the mixed effects likelihood is thus

$$L_{\text{mix}}^n\left(\theta^n \middle| D^n\right) = \left(\frac{e^{-\frac{(\log(\beta^n)-\mu_{\beta,\text{unexp}})^2}{2\sigma_{\beta,\text{unexp}}^2}}}{\sqrt{2\pi}\beta^n\sigma_{\beta,\text{unexp}}}\right)\left(\frac{e^{-\frac{(\log(d_s^n)-\mu_s)^2}{2\sigma_s^2}}}{\sqrt{2\pi}d_s^n\sigma_s}\right)\left(\frac{e^{-\frac{(\log(d_l^n)-\mu_l)^2}{2\sigma_l^2}}}{\sqrt{2\pi}d_l^n\sigma_l}\right)\left(\frac{e^{-\frac{(\log(d_a^n)-\mu_a)^2}{2\sigma_a^2}}}{\sqrt{2\pi}d_a^n\sigma_a}\right)\left(\frac{\left(\rho^n\right)^{\alpha_{\text{unexp}}-1}\left(1-\rho^n\right)^{\beta_{\text{unexp}}-1}}{B\left(\alpha_{\text{unexp}},\beta_{\text{unexp}}\right)}\right)L_{\text{mod}}^n\left(\theta^n \middle| D^n\right)$$

As the proportion of the ASCs that are long-lived must be bounded by 0 and 1, the local parameters ρ^n are assumed to be drawn from a Beta distribution.

And for individuals in the previously exposed group, the mixed effects likelihood is:

$$L_{\text{mix}}^n\left(\theta^n \middle| D^n\right) = \left(\frac{e^{-\frac{(\log(\beta^n)-\mu_{\beta,\text{exp}})^2}{2\sigma_{\beta,\text{exp}}^2}}}{\sqrt{2\pi}\beta^n\sigma_{\beta,\text{exp}}}\right)\left(\frac{e^{-\frac{(\log(d_s^n)-\mu_s)^2}{2\sigma_s^2}}}{\sqrt{2\pi}d_s^n\sigma_s}\right)\left(\frac{e^{-\frac{(\log(d_l^n)-\mu_l)^2}{2\sigma_l^2}}}{\sqrt{2\pi}d_l^n\sigma_l}\right)\left(\frac{e^{-\frac{(\log(d_a^n)-\mu_a)^2}{2\sigma_a^2}}}{\sqrt{2\pi}d_a^n\sigma_a}\right)\left(\frac{\left(\rho^n\right)^{\alpha_{\text{exp}}-1}\left(1-\rho^n\right)^{\beta_{\text{exp}}-1}}{B\left(\alpha_{\text{exp}},\beta_{\text{exp}}\right)}\right)L_{\text{mod}}^n\left(\theta^n \middle| D^n\right)$$

Note that for individuals in the previously exposed group, the pre-existing antibody level A_0^n will be variable, depending on a large number of covariates such as time since previous residence in an endemic area. We therefore do not attempt to constrain pre-existing antibody levels using mixed effects.

Total model likelihood

Denote $D = \left\{D^1, ..., D^N\right\}$ to be the vector of data for all *N* participants. We denote $\theta = \left(\beta_{\text{unexp}}, \beta_{\text{exp}}, d_s, d_l, d_a, \rho_{\text{unexp}}, \rho_{\text{exp}}, \sigma_{\beta,\text{unexp}}, \sigma_{\beta,\text{exp}}, \sigma_s, \sigma_l, \sigma_a, \sigma_{\rho,\text{unexp}}, \sigma_{\rho,\text{exp}}, \sigma_{\text{obs}}, \theta^1, ..., \theta^N\right)$ to be the combined vector of global parameters and local parameters to be estimated. The total likelihood is obtained by multiplying the likelihood for each participant

$$L_{\text{total}}\left(\theta \middle| D\right) = \prod_{n \in N} L_{\text{mix}}^n\left(\theta^n \middle| D^n\right)$$

Markov Chain Monte Carlo parameter update

The model was fitted to the data using Markov Chain Monte Carlo (MCMC) methods. Parameters were updated at each MCMC iteration using a random walk Metropolis-Hastings algorithm with two update stages illustrated below. A′ indicates an attempted update.

1. **Local parameter update.** For each participant *n*:
   - Update local parameters: $\theta^{n\prime} = \left(A_0^{n\prime}, \beta^{n\prime}, d_s^{n\prime}, d_l^{n\prime}, d_a^{n\prime}, \rho^{n\prime} \right)$
   - Calculate updated mixed effects likelihood $L_{\text{mix}}^{n}\left(\theta_n^{\prime} \middle| D_n \right)$
   - Accept the parameter update with probability $\min\left(1, \dfrac{L_{\text{mix}}^{n}\left(\theta_n^{\prime} \middle| D_n \right)}{L_{\text{mix}}^{n}\left(\theta_n \middle| D_n \right)} \right)$
2. **Global parameter update.**
   - Update global parameters:

$$\theta' = \left(\beta_{\text{unexp}}{}', \beta_{\text{exp}}{}', d_s{}', d_l{}', d_a{}', \rho_{\text{unexp}}{}', \rho_{\text{exp}}{}', \sigma_{\beta,\text{unexp}}{}', \sigma_{\beta,\text{exp}}{}', \sigma_s{}', \sigma_l{}', \sigma_a{}', \sigma_{\rho,\text{unexp}}{}', \sigma_{\rho,\text{exp}}{}', \sigma_{\text{obs}}{}' \right)$$

   - Calculate updated total likelihood $L_{\text{total}}\left(\theta' \middle| D \right)$ and the updated prior probability density $P\left(\theta' \right)$
   - Accept the parameter update with probability $\min\left(1, \dfrac{L_{\text{total}}\left(\theta' \middle| D \right) P(\theta')}{L_{\text{total}}\left(\theta \middle| D \right) P(\theta)} \right)$

All updates were attempted with Normal proposal distributions. The MCMC algorithm was implemented in C++. The variances of the proposal distributions were first estimated by performing 1 million MCMC iterations to estimate the variance of the posterior distributions. 20 million MCMC iterations were computed with calibration of acceptance rates using a Robbins-Munro algorithm. All Markov chains were visually examined for appropriate mixing and convergence. Such large numbers of iterations were needed because of the large number of parameters to be estimated. The effective number of iterations was calculated using the effective Size routine in the R library coda and the effective size was checked to be > 1,000 for the global parameters. The MCMC fitting process was repeated multiple times to ensure consistent results and test for lack of convergence.

The model was fitted separately to data from all antigens (total IgG and IgG subclasses) with the parameter estimates presented in Supplementary Table S1.

Supplementary figures

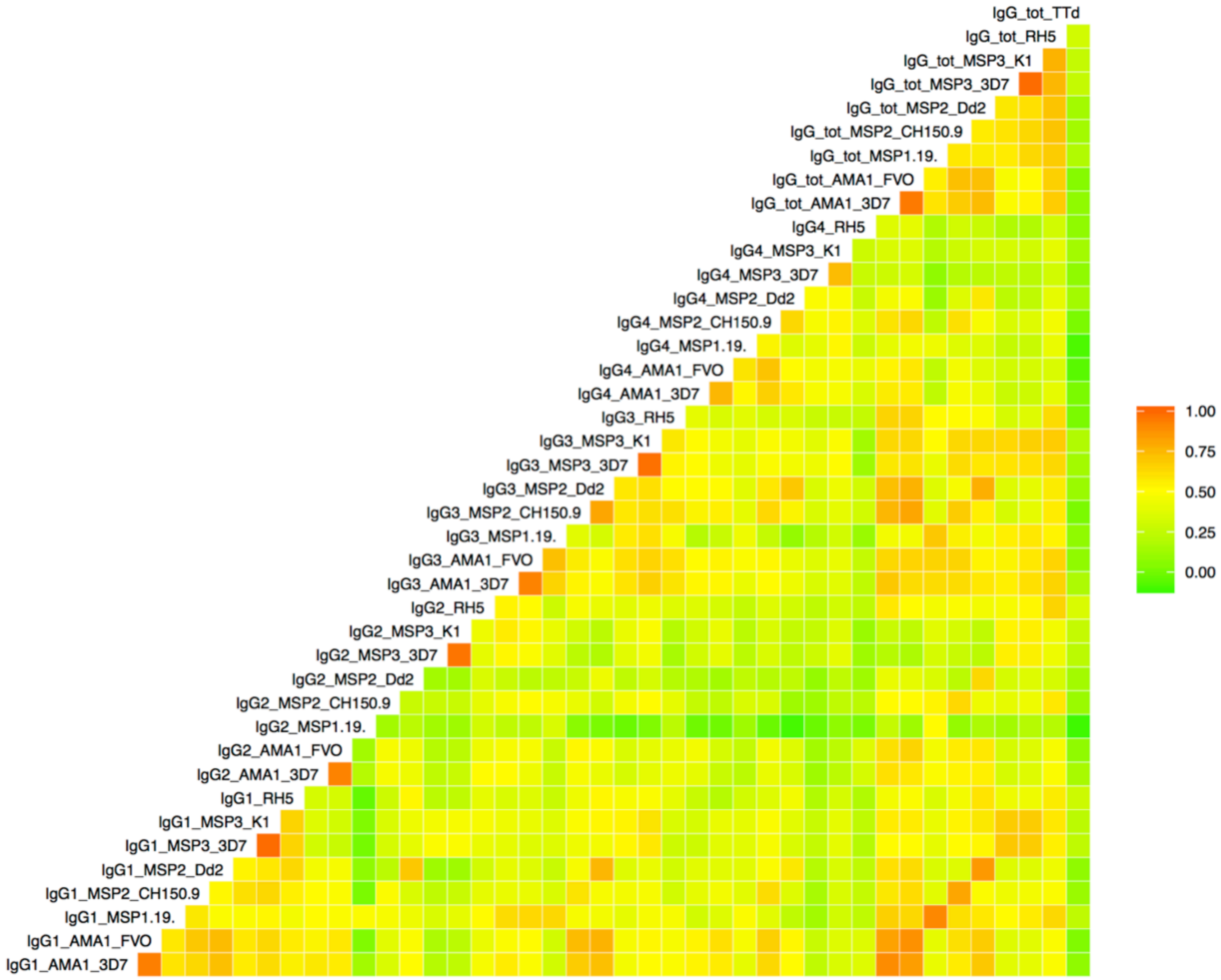

Figure S1. Pairwise correlation in antibody levels. A correlation matrix heat map of all possible pairwise combinations of IgG subclasses and antigen specificities. The magnitude of the correlation is indicated by the colour scale and increases from green to red.

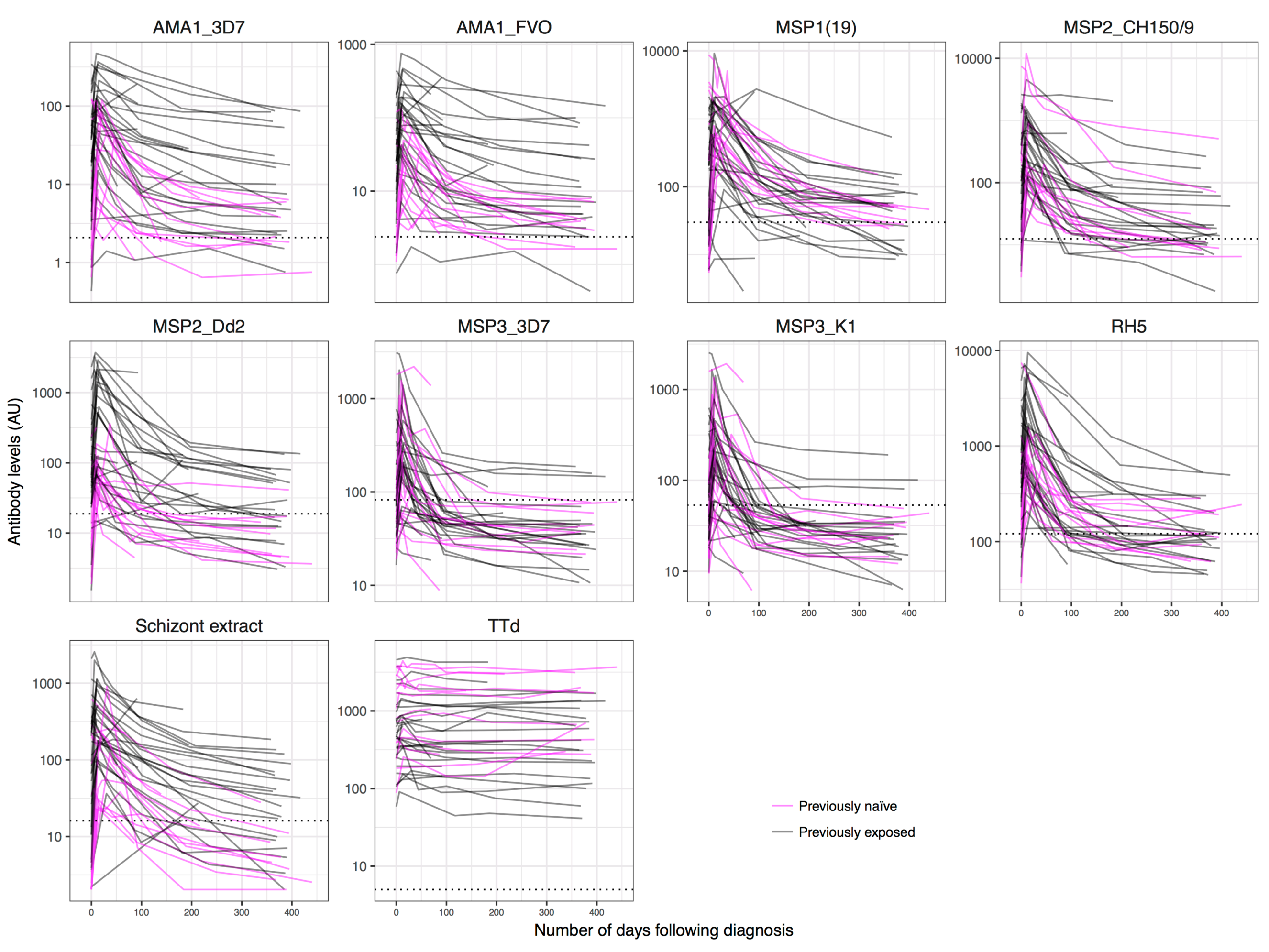

Figure S2. Antigen-specific total IgG responses over time. Individual level antigen-specific total IgG responses to *P. falciparum* recombinant antigens, schizont extract, and TTd over time. Each panel represents the total IgG response to one antigen. Black and magenta coloured lines denote antibody profiles of previously exposed and previously naïve individuals, respectively. The dotted line represents the antigen-specific threshold of seropositivity, defined as the mean+3SD of the reactivity of 20 malaria unexposed negative controls. The antibody levels in arbitrary antibody units are directly comparable only within antigen.

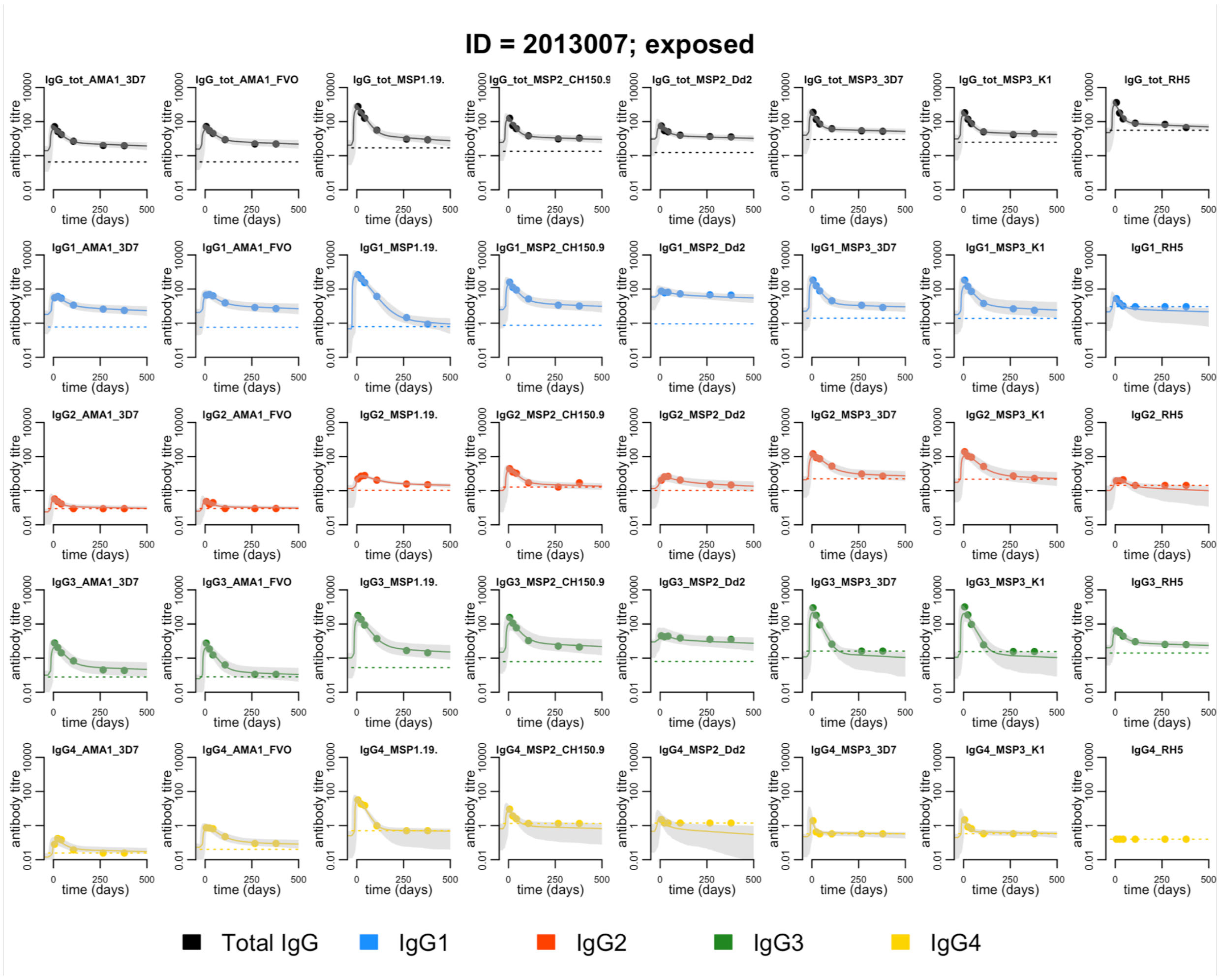

Figure S3. Individual level data and model estimated antibody kinetics for a representative previously exposed study subject (ID: 2013007). Each panel represents one antigen and either total IgG or one IgG subclass. The dots denote the individual sample antibody level in arbitrary units. The solid line denotes the model predicted antibody boost and decay patterns and the shaded grey area the 95% credible interval of the prediction. The dotted line represents the assay lower limit of quantification.

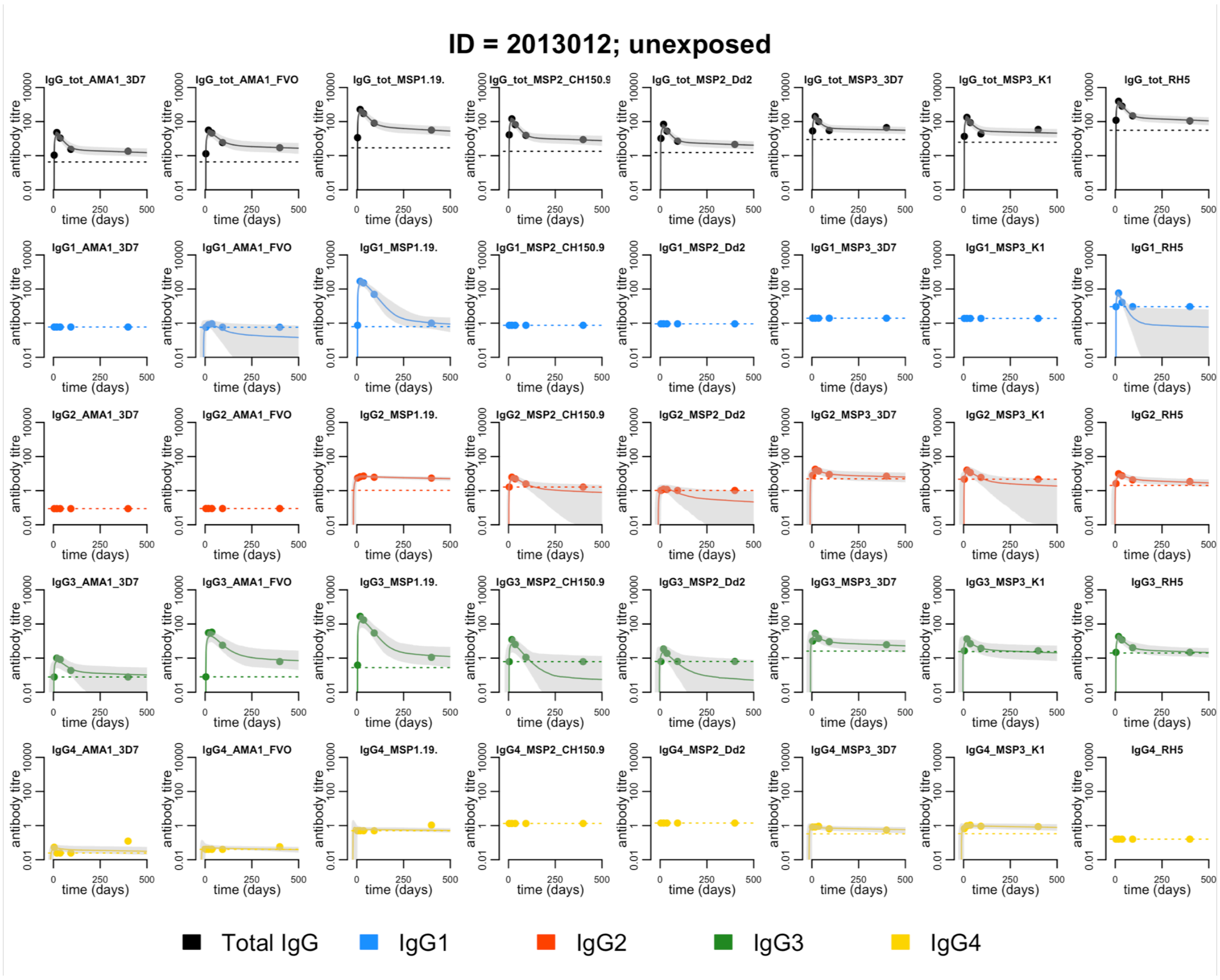

Figure S4. Individual level data and model estimated antibody kinetics for a representative previously naïve study subject (ID: 2013012). Each panel represents one antigen and either total IgG or one IgG subclass. The dots denote the individual sample antibody level in arbitrary units. The solid line denotes the model predicted antibody boost and decay patterns and the shaded grey area the 95% credible interval of the prediction. The dotted line represents the assay lower limit of quantification.

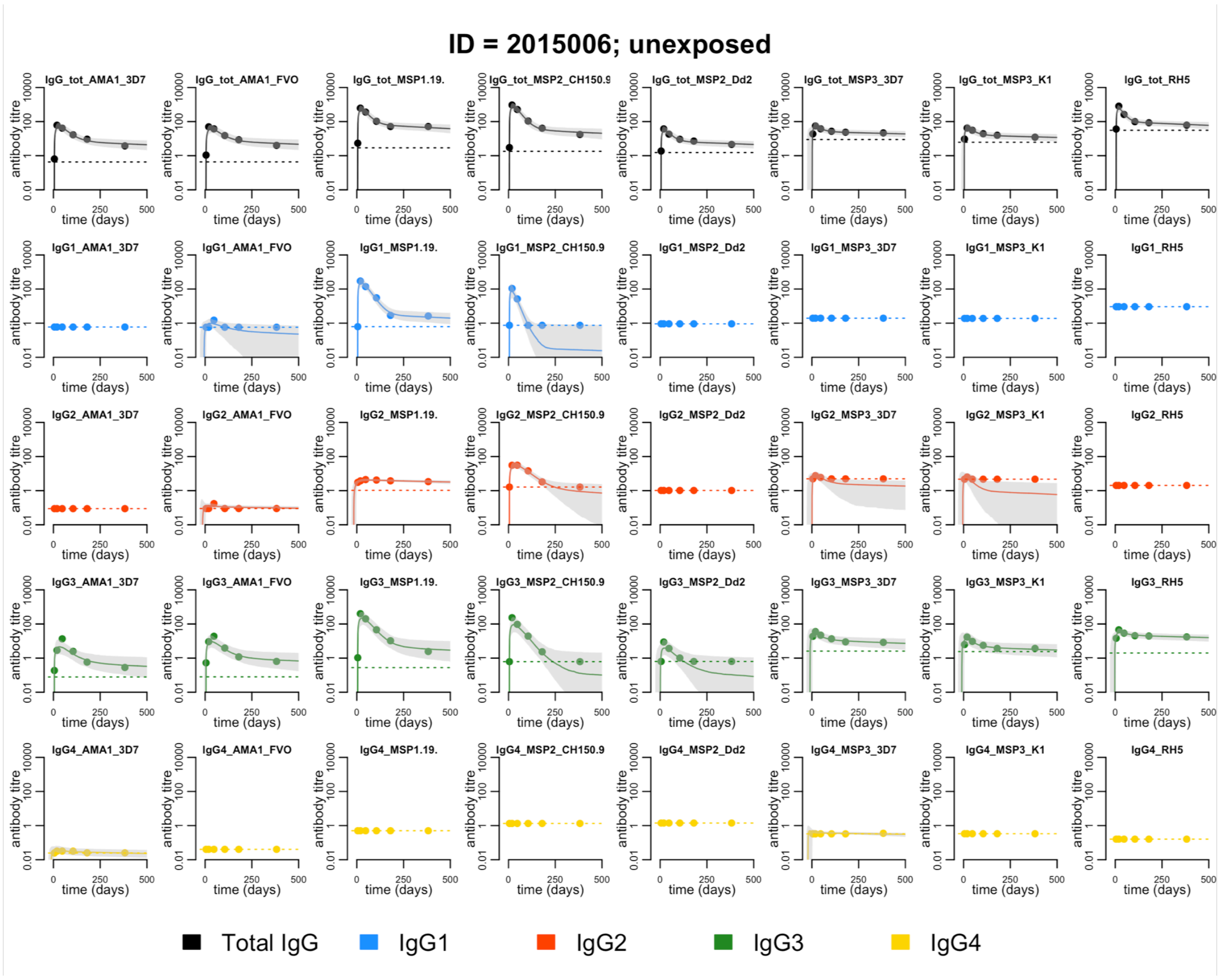

Figure S5. Individual level data and model estimated antibody kinetics for a representative previously naïve study subject (ID: 2015006). Each panel represents one antigen and either total IgG or one IgG subclass. The dots denote the individual sample antibody level in arbitrary units. The solid line denotes the model predicted antibody boost and decay patterns and the shaded grey area the 95% credible interval of the prediction. The dotted line represents the assay lower limit of quantification.

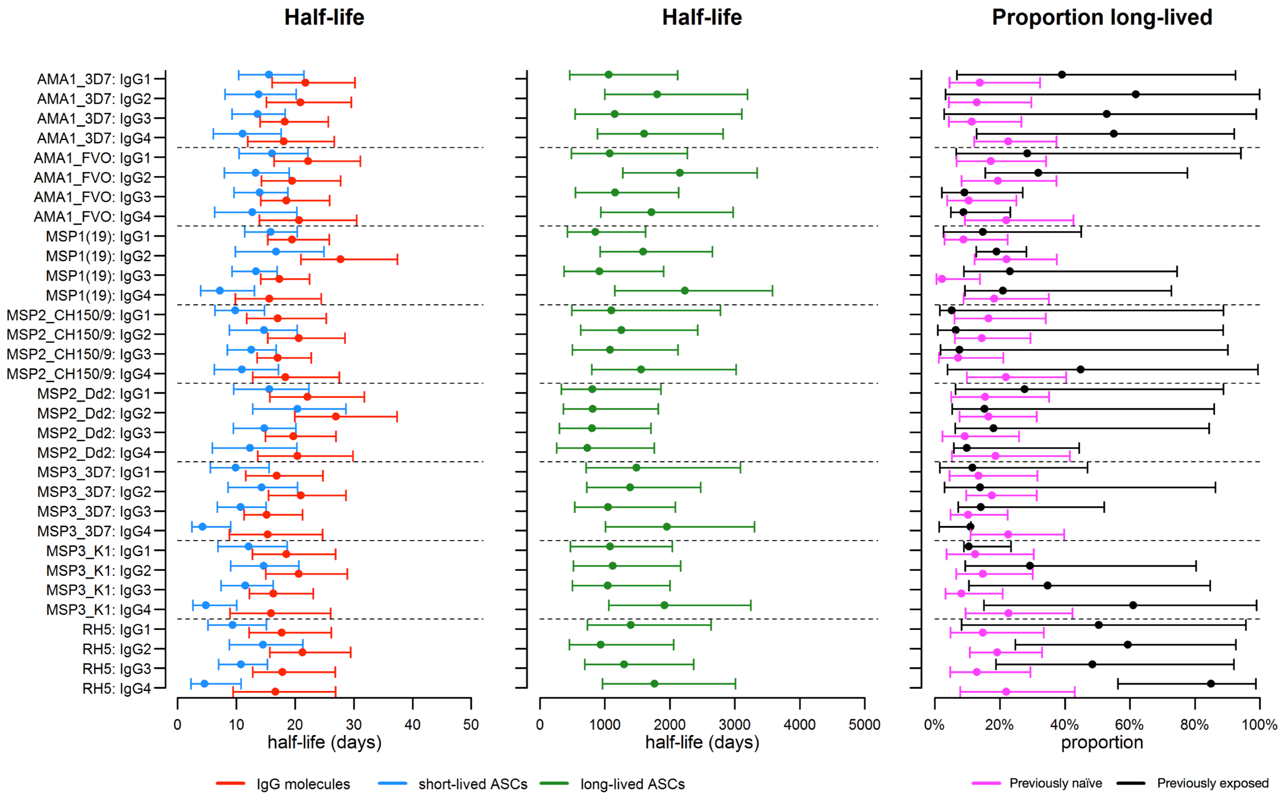

Figure S6. Model population level parameter estimates for the kinetics of the IgG subclass response to each antigen. The dots denote the model parameter estimates and capped error bars the corresponding 95% CrI. The different parameters are indicated by colours: Half-life of antibody molecules (Red), half-life of short-lived ASCs (blue), half-life of long-lived ASCs (green), the proportion of long-lived ASCs in previously naïve (magenta) and in the previously exposed (black). The estimated population level parameters with corresponding variance parameters are also available in supplementary Table S1.
